# Prognostic Scores for Mortality in Invasive Mechanically Ventilated Burn Patients

**DOI:** 10.7759/cureus.63502

**Published:** 2024-06-30

**Authors:** Simone Costa, Catarina Ferros, Ana Reigota, Isabel Tourais, Margarida Marques, João Lopes, Vera Afreixo, Luís Cabral

**Affiliations:** 1 Intensive Care Medicine, Centro Hospitalar de Leiria, Leiria, PRT; 2 Anesthesiology, Centro Hospitalar Tondela Viseu (CHTV), Viseu, PRT; 3 Anesthesiology, Centro Hospitalar Baixo Vouga, Aveiro, PRT; 4 Anesthesiology, Centro Hospitalar e Universitário de Coimbra, Coimbra, PRT; 5 Cardiology, Centro Hospitalar e Universitário de Coimbra, Coimbra, PRT; 6 Mathematics, Center for Research and Development in Mathematics and Applications, University of Aveiro, Aveiro, PRT; 7 Plastic Surgery, Centro Hospitalar e Universitário de Coimbra, Coimbra, PRT

**Keywords:** predicting mortality, intensive burn care, prognostic scores, invasive mechanical ventilation, burn injury

## Abstract

Introduction: Managing burn patients is a challenge requiring a multidisciplinary team with the ability to predict complications and act early to avoid them. There are few studies characterizing the population of critically ill burn patients in need of ventilatory support. This study aimed to describe the population of burn patients in need of invasive mechanical ventilation support and assess in-hospital mortality and the factors associated with it.

Material and methods: A longitudinal retrospective study was conducted, including burn patients admitted to a tertiary hospital burn unit over five consecutive years, who required invasive mechanical ventilation support during their hospitalization. Demographic data, comorbidities, characteristics of the injury scene, etiology, and characteristics of the burn were collected. Length of mechanical ventilation and hospitalization as well as mortality rate were evaluated. The determination of mortality predictors and the prognostic performance of mortality prediction scores were analyzed. A one-year follow-up was performed to evaluate the survival of discharged patients.

Results: A total of 141 patients were included in this study; 68.1% patients were male with a median age of 58 years. The mean percentage of total body surface area (TBSA) burned was 24.5%. Home incidents were the most frequent, and fire was the most common cause of burns (80.9% of patients). The mean Abbreviated Burn Severity Index Score (ABSI) was 7.83, with an area under the curve in receiver operating characteristic curve (ROC) analysis (AUC_ROC) of 0.725; the mean Belgium Outcome of Burn Injury (BOBI) score was 3.45, with AUC_ROC of 0.740 and mean R-Baux of 89.1 and AUC_ROC of 0.834. The mean duration of invasive mechanical ventilation was 16.9±19.3 days. Age (p<0.001), length of mechanical ventilation (p<0.001), length of hospitalization (p<0.001), higher degree of burn (p=0.001), TBSA (p=0.040), and the presence of buttock burn (0.006) were associated with mortality in this sample. In-hospital mortality was 29.8%. The survival group had a 12% death rate at one-year follow-up, mostly in the first three months after discharge.

Conclusion: Age, duration of mechanical ventilation, length of hospitalization, higher degree of burn, TBSA, and the presence of buttock burn were associated with mortality in this sample. R-Baux score was the most accurate test score to predict mortality in this challenging group of patients.

## Introduction

Burn injuries are an under-appreciated type of trauma, responsible for high morbidity and mortality worldwide. A severely burned patient frequently sustains life-threatening injuries, posing a challenge for a multidisciplinary therapeutic approach [1]. Burns can also lead to severe and permanent sequelae and disabilities, affecting physical and mental health, and causing a high burden to patients, their families, and health care systems [2-5].

Literature reports a reduction in burn-associated mortality worldwide, mainly due to advances in intensive care therapy and the development of specialized burn care teams [1,3,4,6,7]. However, besides this reduction, the mortality rate is still high (10-20%) even in well-equipped burn centers [5,8,9]. The most common cause of death in the first 48 hours after burn injury is a unique combination of distributive and hypovolemic shock. After this period, respiratory complications are the most common cause of death in burn victims [9]. Older age, burn size, and the presence of inhalation injury are recognized as the most important factors associated with elevated mortality [5,9-11].

A critical point in the management of burn patients is undoubtedly the evaluation and control of the airway [7,12]. The indication for invasive mechanical ventilation (IMV) is a complex decision and early intubation and the start of mechanical ventilation are frequently required for burn patients [7,11]. There is a substantial increase in the number of burn patients receiving IMV at admission worldwide [12]. These developments seem to be related to aggressive airway management policies of the Advanced Trauma Life Support (ATLS) and international guidelines [6,12,13]. Inhalation injury is both one of the leading indications for mechanical ventilation in burn injury and a leading contributor to prolonged mechanical ventilation [11]. This low threshold for intubation can save lives, but potential complications must be kept in mind that acute respiratory distress syndrome (ARDS) and pneumonia have an increased occurrence during or after mechanical ventilation, making the need for this ventilatory support one of the prognostic factors of mortality in burn patients [5,9-11,14].

Epidemiological studies are crucial to understanding the burn patient's characteristics and outcomes. There are some works reporting overall mortality in burn patients; however, there is still a significant gap in our knowledge regarding the mechanically ventilated ones, in terms of mortality rate, its associated factors, and the applicability of specific mortality scores.

## Materials and methods

This is a retrospective cohort study of burn patients admitted to a specialized burn unit (BU) at a tertiary care hospital in Portugal. Data were retrieved from the hospital’s burns registry from January 2018 to December 2022. The study included 18 years or older patients who required invasive mechanical ventilation (IMV) during the length of stay (LOS). Exclusion factors included patients under 18 years of age, patients with no need for mechanical ventilation, and burn patients who died before reaching the hospital or in the emergency department prior to the transfer to BU.

The following data were collected from patient files: demographic data, comorbidities, characteristics of the injury scene, etiology, and characteristics of the burn, length of mechanical ventilation, need of hemodynamic support at admission to the burn unit, length of hospitalization, mortality rate, together with data for the calculation of Belgium Outcome of Burn Injury Score (BOBI), Abbreviated Burn Severity Index Score (ABSI), and Revised Baux Score (R-Baux). A one-year follow-up was performed to evaluate the survival of discharged patients. Being a retrospective observational study of patients from a suitably anonymized dataset, involving only recording data from the medical record, the ethics committee, according to the Declaration of Helsinki and Council for International Organizations of Medical Sciences (CIOMS) International Ethics Guidelines, waived the need for informed consent.

Data were analyzed using IBM SPSS version 29 software (Armonk, NY: IBM Corp.). Descriptive analysis was presented with absolute frequency (n) and percentage (%) or mean±standard deviation and median (mdn). Two groups were created (G1: survivors; G2: non-survivors). Independent t-test, Mann-Whitney U test, and chi-square test were used to compare those groups in continuous and categorical variables. Odds ratios (ORs) were calculated using logistic regression models for mortality analysis. The predictive value of mortality burn scores in IMV burn patients was evaluated with receiver operating characteristic (ROC) curve with the determination of area under the curve (AUC). Data from a one-year follow-up of G1 group (survivals) was collected and survival analysis was performed with Kaplan-Meier. A p-value less than 0.05 was considered significant.

The primary outcome measure was in-hospital mortality and the predictive accuracy of the burn mortality scores in mechanically ventilated patients. Secondary outcome is to determine prognostic determinants of mortality in IMV burn patients. The tertiary outcome was to assess survival after one year of discharge.

## Results

Characterization of the study population

Between January 2018 and December 2022, 141 burn patients were mechanically ventilated, with an average of 30 IMV burn patients per year, representing 10.2% of the total patients admitted at BU during this five-year period. The years 2020 and 2021 were with more IMV burn patients (n=28 and 31, respectively). Of all patients included, 58.9% (n=83) were transferred from other hospitals, while 41.1% (n=58) were admitted primarily from the hospital's emergency department. The monthly distribution of admissions shows a higher number of admissions in December (n=20, 14.2%).

Demographic Characterization of Study Population

In this study sample, 68.1% (n=96) of burned IMV patients were male. The mean age of patients was 57.6±20.2 years, with a range of 18-99 years. The age distribution showed an increase in the proportion of females to males with age. The average age in females (61.6±20.4 years) was not significantly higher (p=0.107) than males (55.7±19.9 years).

Patient’s Comorbidities

The most frequent comorbidities of patients admitted to this study were analyzed and represented in Table 1.

**Table 1:** Most frequent comorbidities of burned patients submitted to IMV. The data has been represented as number of cases (n) and percentage. COPD: chronic obstructive pulmonary disease; IMV: invasive mechanical ventilation

Comorbidities	n (%)
Arterial hypertension	43 (30.5)
Dyslipidemia	30 (21.3)
Diabetes	28 (19.9)
Obesity	22 (15.6)
Psychiatric pathology	22 (15.6)
Alcoholism	16 (11.3)
Active smoking	15 (10.6)
COPD	10 (7.1)
Arterial fibrillation	7 (5.0)
Drug addiction	7 (5.0)
Stroke history	6 (4.3)
Dementia	5 (3.5)
Epilepsy	4 (2.8)
Heart failure	4 (2.8)
Renal failure	2 (1.4)
Hepatic steatosis	1 (0.7)

Burn characterization

Burn Etiology

Burns etiology is represented in Table 2, with thermal burn caused by fire being the major cause (n=114, 80.9%). Regarding the circumstance of injury, 63.8% (n=90) of burns occurred at home while 24.8% (n=35) were work-related injuries. Burns caused by forest fire injuries accounted for 2.1% (n=3). Self-inflicted or attempted suicides represented 8.5% and aggression in one case (0.7%). Neither the etiology nor the circumstance of injuries have significantly changed over the years.

**Table 2:** Patient characteristics and comparison between survivors vs non-survivors. Age, length of stay, TBSA, and ventilation period data have been represented as mean±SD. The remaining data have been represented as number of cases (n) and percentage. Mann-Whitney U test was used in TBSA analysis (no normal distribution). P-value<0.05 was considered significant. TBSA: total body surface area; IQR: interquartile range; mdn: median

Variable	All episodes, (n=141)	Survivors (n=99)	Non-survivors (n=42)	p-Value
Age (years), mean±SD	57.6±19.8	53.5±19.6	67.2±18.5	<0.001
<20, n (%)	2 (1.5)	2 (2.0)	0	
20-39, n (%)	29 (20.5)	25 (25.2)	4 (9.6)	
40-59, n (%)	39 (27.6)	32 (32.3)	7 (16.6)	
60-79, n (%)	48 (34.0)	29 (29.2)	19 (45.2)	
≥80, n (%)	23 (16.3)	11 (11.1)	12 (28.5)	
Gender				
Male, n (%)	96 (68.1)	71 (71.7)	25 (59.5)	0.155
Female, n (%)	45 (31.9)	28 (28.3)	17 (40.5)	
Length of stay (days), mean±SD	31.0±26.8	32.3±26.4	27.7±27.8	0.351
TBSA%, mean±SD	24.5±23.9 (mdn: 15.0%; IQR: 25.5)	16.7±15.8 (mdn: 12.0%; IQR 15.5)	42.9±29.3 (mdn: 35.0%; IQR 46.8)	(U=3321; p <0.001)
<20%, n (%)	81 (57.4)	71 (71.7)	10 (23.8)	
20-39%, n (%)	30 (21.3)	18 (18.2)	12 (28.6)	
40-59%, n (%)	16 (11.3)	8 (8.1)	8 (19.0)	
60-79%, n (%)	2 (1.4)	1 (1.0)	1 (2.4)	
≥80%, n (%)	12 (8.5)	1 (1.0)	11 (26.2)	
Burn degree				
2nd, n (%)	36 (25.5)	35 (35.4)	1 (2.4)	<0.001
2nd and 3th, n (%)	93 (66.0)	60 (60.6)	33 (78.6)	
3th, n (%)	12 (8.5)	4 (4.0)	8 (19.0)	
Inhalation injury, n (%)	61 (43.3)	41 (41.4)	20 (47.6)	0.496
Broncofibroscopy, n (%)	90 (63.8)	69 (68.7)	22 (52.4)	-
Vasopressor support at admission, n (%)	39 (27.7)	24 (24.2)	15 (35.7)	0.164
Ventilation period, days, mean±SD	17.2±19.8	15.3±18.4	22.0±22.4	0.067
Burn injury mechanism				
Thermal (fire), n (%)	114 (80.9)	81 (81.8)	33 (78.6)	0.735
Thermal (liquid/contact), n (%)	13 (9.2)	8 (8.1)	5 (11.9)	
Thermal (explosion), n (%)	1 (0.7)	1 (1.0)	0	
Electrical, n (%)	11 (7.8)	7 (7.1)	4 (9.5)	
Type of accident				
Domestic, n (%)	90 (63.8)	57 (57.6)	33 (78.6)	0.085
Work-related, n (%)	35 (24.8)	31 (31.3)	4 (9.5)	
Suicide attempt, n (%)	12 (8.5)	8 (8.1)	4 (9.5)	
Forest fire, n (%)	3 (2.1)	2 (2.0)	1 (2.4)	
Aggression, n (%)	1 (0.7)	1 (1.0)	0	

Burn Anatomical Localization

Regarding the anatomical location of the burn, injuries usually affect different body segments simultaneously, with head and neck being the most common (n=107, 75.9%), followed by the upper limbs and the thorax. Sixty-one burn patients had airway damage confirmed by bronchoscopy, representing 43.3% of the sample. The anatomical distribution of burn lesions is represented in Table 3.

**Table 3:** Anatomical location of burn of mechanically ventilated patients. The data has been represented as number of cases (n) and percentage.

Anatomical location	Head	Upper limbs	Anterior and/or posterior thorax	Airway lesion	Lower limbs	Buttocks	Perineum	Abdomen
n (%)	107 (75.9)	84 (59.6)	75 (53.2)	64 (42.4)	48 (34.0)	20 (14.2)	18 (12.8)	17 (12.1)

Burn Extension

The mean and median percentage of total body surface area of burn (TBSA%) were 24.5±23.9% and 15.0%, respectively, with range of 1-100%. TBSA was classified into five groups (Table 2), with TBSA under 20% being the most representative (57.4%). There were no significant differences in mean TBSA between genders (male: 24.8±24.6% vs female: 23.7±22.6%, p=0.800) or between age categories (chi-square=550.12, df=536, p=0.327). The distribution of TBSA% is similar between the different circumstances of injury (chi-square=175.46, df=268, p=1.000) and did not change with the presence of inhalatory lesions (inhalatory lesion present: mean 28.1±25.4%; inhalatory lesion absent: 22.7±22.4%; p=0.112).

Airway lesions, hemodynamic instability, and mechanical ventilation

Bronchoscopy was performed in 90 patients (63.8%), with identification of airway burns in 61 patients (43.3%). Thirty-nine patients (27.7%) were hemodynamically unstable upon admission to the burn unit, with a need for vasopressor support. Eighty-three patients initiated invasive mechanical ventilation (IMV) before admission to the burn unit (58.9%). Of those, 43.3% (n=36) were extubated in the first 48 h after admission. Fifty-eight patients (41.1%) needed to start IMV after admission to the BU. The main reasons for intubation were the suspicion of airway injury due to the circumstances/location of the burn, as well as the presence of respiratory failure/ARDS due to systemic inflammatory response, with no response or no indication for non-invasive mechanical ventilation trial. The mean and median length of IMV were 17.2±19.8 days and eight days, respectively, with a range of 1-95 days. The mean and median LOS for burned IMV patients were 31.0±26.8 and 23.0 days, respectively, with a range of 2-127 days. There were no significant differences in the number of days of IMV between genders (male: mean 15.4±17.0 days vs female: 20.3±23.3 days, p=0.166). The mean length of IMV was similar between patients with and without inhalation burn (19.2 vs 15.8 days, respectively) (p=0.319). Thirty ventilated patients (21.3%) had infectious episodes (mostly respiratory and at the wound site), requiring antibiotic therapy.

Primary outcome: mortality

Observed Mortality

The overall in-hospital mortality in this study was 29.8% (n=42). From burn-surviving IMV patients (n=99), 29.1% were transferred to other hospitals to maintain care, 26.2% were discharged home, 2.1% were discharged to a nursing home/rehabilitation center, 9.2% were transferred to the plastic surgery department, and 3.5% were transferred to non-specialized wards.

Mortality Comparison Between Survivors and Non-survivors

The comparison between G1 and G2 is presented in Table 2. Most deceased patients were male (59.5%) and there were no significant differences in mortality between genders (p<0.155). The mean age of deceased patients was 67.2±18.5 years, with a range of 21-99 years. There is a statistically significant association between age and mortality, with an increase in mortality with age, especially over 50 years (p<0.001).

The causes of burns in deceased patients are represented in Table 2, showing that thermal burns due to the fire were responsible for 78.6% of the deaths (n=33), followed by contact/high-temperature liquid and electrical burns. The mean TBSA% of burns in deceased patients (42.9±29.3%) was significantly higher (p<0.01) than in non-deceased patients (16.7±15.7%).

Twenty-two of the non-survivors (52.4%) were intubated and ventilated at admission at the BU correlating with the percentage of deceased patients with airway burns (n=20, 47.6%). This subgroup was submitted to invasive mechanical ventilation during a mean of 22.0 days (median 13.5, range of 2-94 days), without significant difference (p=0.067) from those non-deceased (mean 15.3 days).

At admission to BU, 35.7% (n=15) of non-survivor patients were hemodynamically unstable, with the need for vasopressor support. There were no significant differences in the length of stay between survivors and non-survivor patients (32.3±26.4 vs 27.7±27.8 days, p=0.351).

Regarding mortality prediction scores in G2 group, the mean BOBI score was 4.9±2.4 (mean predicted mortality was 37.5±31.4%), mean ABSI was 9.48±3.2 (predicted mortality was 30-50%), and mean revised-Baux score of 117.4±30.9 (predicted mortality was 100%).

Secondary outcome: mortality determinants

Chi-square and t-tests were performed in univariate analysis to evaluate variables with possible association with mortality. Gender, need of vasopressor support at admission, intubation at admission, cause of burn, type of accident, anatomic area of burn in the head or upper limbs, length of mechanical ventilation, and length of hospitalization stay were similar between the groups. Also, comorbidities like arterial hypertension, dyslipidemia, obesity, chronic obstructive pulmonary disease (COPD), diabetes, psychiatric pathology, alcoholism, active smoking, arterial fibrillation, drug addiction, stroke history, heart failure, epilepsy, renal failure, and hepatic steatosis were not statistically different between the two groups.

By this univariate analysis, statistically significant differences between the two groups were found for age (p≤0.001), TBSA (p≤0.001), comorbidities of dementia (p=0.028), need of antibiotic therapy during length of stay (p=0.027), and degree of burn (p≤0.001). The anatomical site of burn was significantly different between survivors and non-survivors in lower limbs (p≤0.001), thorax (p≤0.001), abdomen (p≤0.001), perineum (p≤0.001), and buttocks (p≤0.001).

A logistic regression analysis was performed including the variables with significant results in univariate analysis to avoid possible confounders regarding mortality association. In this multivariate analysis the model that predicts 90.8% of cases included length of IMV and hospitalization by stepwise selection technique. Results are shown in Table 4.

**Table 4:** Factors associated with mortality based on logistic regression analysis. P-value<0.05 was considered significant. Hosmer-Lemeshow p=0.955; R^2^ Cox-Snell p=0.525; R^2^ Nagelkerke p=0.746. LOS: length of stay in burn unit; TBSA: total body surface area; OR: odds ratio; EXP (B): exponentiated coefficient

Variables	B	SE	Wald	df	Sig.	ORs	95% CI for EXP (B)	
							Lower	Upper
Age	0.081	0.022	13.297	1	<0.001	1.084	1.038	1.133
Length of mechanical ventilation	0.102	0.029	12.766	1	<0.001	1.108	1.054	1.199
LOS	-0.130	0.032	16.153	1	<0.001	0.878	0.825	0.936
Higher degree of burn	5.345	2.117	6.373	1	0.012	209.528	3.304	13207.608
TBSA%	0.051	0.025	4.224	1	0.040	1.053	1.002	1.105
Burn in buttocks	4.345	1.585	7.512	1	0.006	77.090	3.449	1723.303
Constant	-21.228	8.710	6.891	1	0.002	0.000	-	-

A strong association with in-hospital mortality was found for six variables in this sample: age, length of mechanical ventilation, length of stay, third-degree burns, TBSA, and the presence of buttock burns. From those, the presence of third-degree burns contributed to higher odds of mortality (OR=209.52, p=0.012), followed by burns on the buttocks (OR=77.090, p=0.006). It is to be noted that almost all deceased patients (n=41) had third-degree burns.

For every year increase in age, the odds of dying during hospitalization were increased by 1.084. The odds of dying during hospitalization increased by 1.1 and 0.9 times for each day spent on mechanical ventilation and hospitalization, respectively. As expected, the odds of dying was greater with higher TBSA with an OR of 1.053 (p=0.040). Ventilated patients with burns on the buttocks had 77 times greater odds of death (p=0.006).

Predictive Value of Burn Scores in Mechanically Ventilated Patients

A determination of the values from three different burn mortality scores (BOBI, ABSI, and R-Baux) for both groups of patients was done and its results are presented in Table 5.

**Table 5:** Burn mortality scores. P-value<0.05 was considered significant. BOBI: Belgium Outcome of Burn Injury Score; ABSI: Abbreviated Burn Severity Index Score; R-Baux: Revised Baux Score

Variable	All episodes, (n=141)	Survivors (n=99)	Non-survivors (n=42)	p-Value
BOBI (mean±SD)	3.45±2.2	2.84±1.9	4.9±2.4	<0.001
Predicted mortality (%)	21.5%	14.6%	37.5%	<0.001
ABSI score (mean±SD)	7.83±2.8	7.13±2.3	9.48±3.2	<0.001
Predicted mortality (%)	20-50%	10-20%	30-50%	
Baux score (mean±SD)	89.2±33.1	77.2±26.0	117.4±30.9	<0.001
Predicted mortality (%)	82.35%	48.83%	100%	

**Figure 1:**
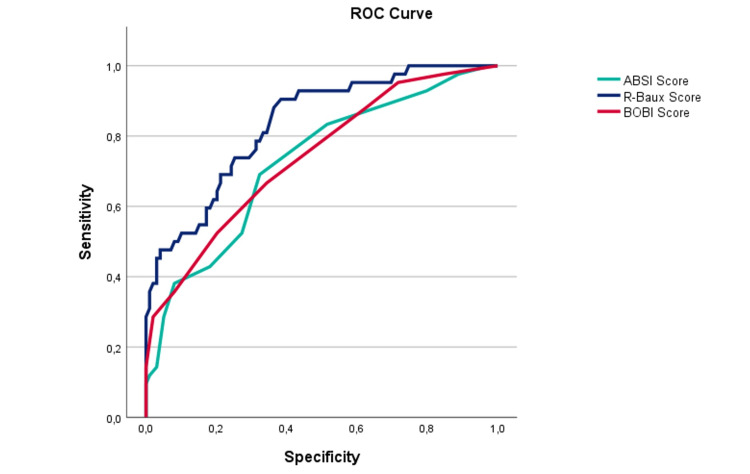
ROC curve for buns scores applied to the study population. ROC: receiver operating characteristic curve; AUC: area under the curve; BOBI: Belgium Outcome of Burn Injury Score; ABSI: Abbreviated Burn Severity Index Score; R-Baux: Revised Baux Score

ABSI and BOBI scores appeared to be good predictors of mortality in this sample; however, R-Baux showed excellent discriminatory capacity (AUC: 0.834) and as such is the most appropriate score for mechanically ventilated burn patients (Table 6 and Figure 1).

**Table 6:** ROC analyses for burn scores applied to the study population. ROC: receiver operating characteristic curve; AUC: area under the curve; BOBI: Belgium Outcome of Burn Injury Score; ABSI: Abbreviated Burn Severity Index Score; R-Baux: Revised Baux Score

Score mortality	AUC	Asymptotic 95% confidence interval	
		Lower bound	Upper bound
ABSI score	0.725	0.633	0.817
BOBI score	0.740	0.651	0.829
R-Baux score	0.834	0.763	0.905

Tertiary outcome: one-year follow-up of survivors

**Figure 2:**
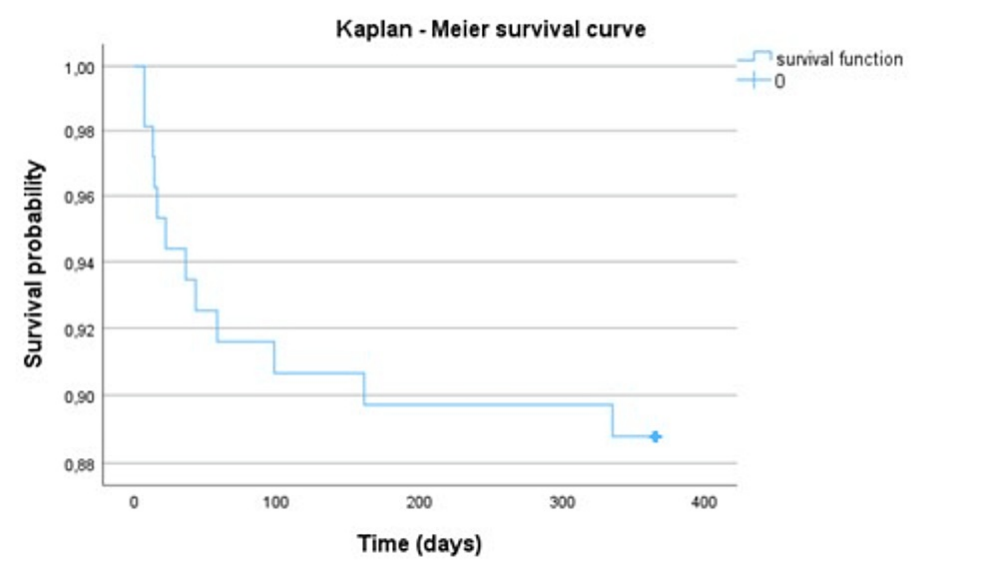
Cumulative survival probability in one-year follow-up of mechanically ventilated burns survival patient.

The one-year follow-up of survivor patients was analyzed. Of the 99 patients included, 12.1% were dead in a period of one year (n=12) with 58.3% (n=7) being males. The mean age of deceased patients during one-year follow-up was 68.58±21.22 years. The average duration of invasive mechanical ventilation in the group of patients who died was 34.4±21 days. As demonstrated in Kaplan-Meier Curve, most deaths occurred in the first three months after discharge from the burn unit (n=9, 90%), with a mean of 67.50±95.7 days after (median: 29 days, range: 7-328 days after discharge) (Figure 2).

## Discussion

The burn unit is a referral center, so it would be expected to find a large percentage of patients transferred from other hospitals, as is the case of this sample, where approximately 60% of patients came from other facilities. In this study, it was found that the BU treated approximately 30 IMV burn patients per year, corresponding to 10.2% of all admitted patients. This number is inferior to those reported in other studies, with the percentage of IMV patients ranging from 22% to 26.9% [5,9,11,15]. This result could be explained by the fact that, even though Portugal is a small country, there are five burn centers, so the patients are distributed to the centers according to their location/residency, therefore diluting the number of IMV patients, differently from other studies where the majority of patients are admitted in one center. The years with the highest number of ventilated patients were 2020 and 2021. From the retrieved data it is not clear how the COVID pandemic might have influenced these results; however, during that period the BU admitted some patients with burns and simultaneous coronavirus infection, complicating their prognosis and clinical condition with progression to ARDS, resulting in a greater need for ventilatory support.

There was a higher incidence of burns requiring IMV in December, different from the results reported by Bartosch et al. who reported more burns in summer and autumn [1]. This is most probably due to an increase of domestic burns by fire during the cold weather season, mostly affecting older and/or handicapped people falling over fireplaces, in a region with low income, where bonfires are still frequently used for warming houses, especially in the interior of the country. The higher percentage of male patients included in the study did not reach statistical significance as verified in previous studies [1,3,5,9,16-20].

The patients included in this sample had an average age of 57.6 years, a little higher than that reported in other studies [16,18,19]. In line with Bartosch et al. and Mcgill et al., females in need of IMV were older; however, without a statistically significant difference in men's age [1,21]. This higher age could be explained by the aging of the Portuguese population in the last decades and by the higher life expectancy of women, conditioning the presence of comorbidities that may facilitate the emergence of accidents with burns. Also, higher age is associated with an increase in comorbidities and a higher risk of organ failure with the need for mechanical ventilation [22]. Although the present study focused on a group of patients with IMV, without comparison with those without IMV, it is highly conceivable the hypothesis that the age distribution of burn patients requiring IMV follows the one observed in the global population of hospitalized patients.

Regarding the associated comorbidities, the most frequent were high blood pressure, dyslipidemia, and diabetes, following the same distribution of comorbidities in the general population [2]. Psychiatric pathology and alcoholism were present in 15.6% and 11.3%, respectively, and might contribute to the presence of 8.5% of self-inflicted burns/suicide attempts observed in this sample. The main etiology of burns in patients who were later subjected to mechanical ventilation was the attainment by flames, whether due to a domestic accident or a forestry accident, similar to what is found in the literature [1,9,18,19].

The average TBSA% of burn in this sample was approximately 24.5% (median: 15.0%) within a wide range (from 1% to 99%). This value is linked to a great risk of developing a systemic inflammatory response, leading to the emergence of ARDS and the need for IMV. Moreover, the greater the TBSA, the longer the length of hospital stay, leading to the emergence of complications such as nosocomial pneumonia, which may also lead to a greater risk of needing ventilatory support. The mean TBSA observed in this study is similar to those found in the literature [15,18,19]. However, the majority of patients included in this study had a TBSA lower than 20%. In fact, other factors, such as the suspicion of burn/airway injury, contributed to the need for ventilatory support, regardless of the extent of the burn, as seen in this sample, with the absence of a statistically significant difference in the TBSA in patients with or without inhalation injury. The somewhat unusual higher percentage of patients presented with burns in the head and cervical region in this sample, probably explains the endotracheal intubation that sometimes was superfluous and unnecessary, leading to the start of invasive mechanical ventilation before admission to the BU (corresponding to 58.9% of the sample patients), according to national and international guidelines in the case of suspected airway compromise [6,11,13]. However, 43% of the patients admitted already under IMV, were extubated within the first 48 h due to the absence of confirmation of airway burns by bronchoscopy and/or good clinical evolution without the need for ventilatory support. This result is in line with that described by Cachafeiro et al. and Orozco-Peláez which indicated that we are possibly being too invasive in pre-hospital airway approach in burn patients [12,17].

ARDS resulting from the systemic inflammatory response caused by the burn itself and by its infectious complications contributed to the remaining 41.1% who started IMV in the burn unit. The average duration of ventilation was approximately 17 days, with a range of 1-95 days, higher than reported in the literature, ranging from four to 11 days [9,10,20]. This fact may be related to the elevated age of the sample population, as increasing age is associated with difficulty weaning from ventilation and increased IMV length. Despite the higher number of days under IMV, the length of stay was similar to those reported in the literature, being approximately 31 days, with a range of two to 129 days [15,20,23]. Airway burns were confirmed in a high percentage of patients (43.3%); however, contrary to what was described in most other studies, the occurrence of airway burns did not show a relation with mortality in this sample [1,11,15,18]. It should be highlighted that those studies include both mechanical and non-mechanically ventilated patients, and their results are different from those studies that included IMV burn patients only, like the present one, in which inhalatory lesion was not associated with higher mortality [5,17]. Likewise, in our study, there was no difference in the number of days of IMV and length of hospitalization in patients with and without inhalation injury (p=0.319 and p=0.456, respectively).

The average duration of IMV in non-survivor patients was longer than the one observed in survivors. After performing logistic regression with forward stepwise methodology, both the duration of invasive mechanical ventilation and the length of hospitalization were included, showing an association with mortality after eliminating confounding variables. From the six predictors of in-hospital mortality identified in this sample (age, length of mechanical ventilation, length of hospitalization, third-degree burns, TBSA, and the presence of buttock burns), the presence of third-degree burns had a higher impact on mortality (OR=209.6; p=0.012), followed by the presence of burns in the buttocks (OR=77.09, p=0.006). The first five predictors of mortality were also described in previous studies [1,3,5,10,15,20,23]. From the authors’ knowledge, no studies showed an association between buttocks burns and higher mortality. It is plausible that buttocks burns might be associated with a higher incidence of pressure ulcers and wound infections, paying attention to the lack of mobility of sedated ventilated patients and their complicated positioning, delaying wound healing, and also to the potential emergence of infection episodes due to the proximity to so-called dirty anatomical areas, that may evolute to sepsis, substantially increasing the mortality. However, more directed studies must be carried out to confirm this hypothesis.

Obesity is a known prognostic factor for mortality in the global IMV population; however, this was not observed in this study, where the association between obesity and mortality in IMV burn patients was not statistically significant. This data is aligned with data published by Xiao et al. [18]. Nevertheless, it is possible that the relatively small number of obese burn-ventilated patients in this sample might have biased this result. In the same way, this study does not confirm the result of Santos et al., as chronic obstructive pulmonary disease (COPD) was not found to be a prognostic factor of mortality [22].

In line with the literature, the R-Baux score was the one that best-predicted mortality in this sample of mechanically ventilated burn patients [5,20,24]. The survivor’s follow-up of one year showed that 12% of the discharged patients died during this period. The majority of those deaths occur during the first three months after discharge from the BU, as observed in the Kaplan-Meier curve of cumulative survival, a result like those found in other studies [15,25].

This study has some limitations that should be considered. First, this is a single-center retrospective observational study conducted in a university hospital with a small sample; thus, the interpretation and extrapolation of our results to other IMV burn patients should be made with caution. The design of the study only included burned IMV patients; therefore, comparisons between ventilated and non-ventilated patients were not made and extrapolation of the results for non-ventilated patients may not be done. The diagnosis of inhalation injury may be underestimated because, despite bronchoscopy being performed in most of the patients, some missed it. The study did not include other scores for assessing organ dysfunction and prognosis in critically ill patients, such as Acute Physiology and Chronic Health Evaluation (APACHE II), Sequential Organ Failure Assessment (SOFA), and Therapeutic Intervention Scoring System (TISS-28), since they were not available at the time of data collection and some of them are not yet validated for burn patients. Adding organ dysfunction and prognosis scores to specific burn scores possibly could have given a more accurate evaluation of the prognostic of these burned IMV patients.

## Conclusions

This single-center study, with a sample of burn patients under mechanical ventilation, demonstrated an intra-hospital mortality of 29.8%, with the identification of the following six mortality predictors: age, duration of mechanical ventilation, length of hospitalization, higher degree of burn, percentage of TBSA and the presence of buttocks burns. The R-Baux score proved to be the best predictor of mortality in this population. In the group of burns survivors subjected to invasive ventilation, mortality was higher in the first three months of follow-up after hospital discharge, being around 12% at 12 months. In the future, prospective multicentric studies in invasive mechanically ventilated burn patients are needed and could make a definite validation of these conclusions.
